# Accommodating Species Climate-Forced Dispersal and Uncertainties in Spatial Conservation Planning

**DOI:** 10.1371/journal.pone.0054323

**Published:** 2013-01-22

**Authors:** Priscila Lemes, Rafael Dias Loyola

**Affiliations:** 1 Programa da Pós-graduação em Ecologia e Evolução, Universidade Federal de Goiás, Goiânia, Goiás, Brazil; 2 Departamento de Ecologia, Universidade Federal de Goiás, Goiânia, Goiás, Brazil; Indiana University, United States of America

## Abstract

Spatial conservation prioritization should seek to anticipate climate change impacts on biodiversity and to mitigate these impacts through the development of dynamic conservation plans. Here, we defined spatial priorities for the conservation of amphibians inhabiting the Atlantic Forest Biodiversity Hotspot that overcome the likely impacts of climate change on the distribution of this imperiled fauna. First, we built ecological niche models (ENMs) for 431 amphibian species both for current time and for the mid-point of a 30-year period spanning 2071–2099 (i.e. 2080). For modeling species' niches, we combined six modeling methods and three different climate models. We also quantified and mapped model uncertainties. Our consensus models forecasted range shifts that culminate with high species richness in central and eastern Atlantic Forest, both for current time and for 2080. Most species had a significant range contraction (up to 72%) and 12% of species were projected to be regionally extinct. Most species would need to disperse because suitable climatic sites will change. Therefore, we identified a network of priority sites for conservation that minimizes the distance a given species would need to disperse because of changes in future habitat suitability (i.e. climate-forced dispersal) as well as uncertainties associated to ENMs. This network also maximized complementary species representation across currently established protected areas. Priority sites already include possible dispersal corridors linking current and future suitable habitats for amphibians. Although we used the a top-ranked Biodiversity Hotspot and amphibians as a case study for illustrating our approach, our study may help developing more effective conservation strategies under climate change, especially when applied at different spatial scales, geographic regions, and taxonomic groups.

## Introduction

A wide range of evidences indicate climate change as one the greatest threats to biodiversity in the 21^th^ century [1]. Climate change impacts, which may have already resulted in several recent species extinction [2], are species-specific and produce shifts in species phenology, ecological interactions, and geographical distributions [3]–[4]. Global climate change poses new challenges to biodiversity conservation especially because it induces species range shifts yielding additional complexity and uncertainty to definition and implementation of spatially oriented actions for conservation investment [5]. Here we address this challenge by developing spatial conservation plans that consider the likely species' range shifts under baseline and future climate scenarios.

Climate change effects on biodiversity depend on how species' distribution will respond to such changes. These responses are usually inferred trough ecological niche models (henceforth ENMs) [6]. Currently, there are several methods for modeling species occurrences as a function of environmental variables, which is the standard approach used by ENMs (see Franklin [7] and Peterson et al. [8], for recent reviews). Techniques for generating ENMs range from very simple bioclimatic envelope models up to complex machine learning-based methods [7]. However, model uncertainty arise from many sources, such as the methods and the climate projections used to generate ENMs [9], not to mention the limitations to model extrapolation in space and time (i.e. model transferability), and how to evaluate model performance [10].

The ongoing debate on model performance, statistical fit, and transferability indicates that it is still difficult to determine the best method for modeling species' ecological niche, because the outcome of these methods is strongly dependent on data availability and geographic scale for which they have been projected [11]. To cope with these issues, a combination of different projections built upon different climatic conditions and modeling methods – the ensemble forecasting approach – has been suggested as more conservative than single model analysis [9]. Ensembles of forecasts should be used when it is impossible to determine which type of model should produce most accurate predictions. Predictions from multiple models or from multiple input data sets are usually averaged and weighted by model accuracy. Thus, by combining different model projections, final consensus may benefit from accurate models, although depending on how model predictions are combined, poor model predictions may cancel accurate models [12]. When applying ensemble of forecasts the final solution is an unique consensus, weighted by overall statistical fit (e.g. TSS statistics, AUC values) of combined models, from which is also possible to quantify and map model uncertainties [13].

Ecological niche models can be useful to develop conservation plans, especially in regions where complete information on species distribution is not available or will not be in the future, as expected in megadiverse countries [14]. Other studies have used ENMs for conservation planning, however ENM uncertainties are rarely incorporated (but see Carroll et al. [15], Wilson [16], for recent examples). Therefore, it is still necessary to develop science-based portfolios of spatial priorities in which species' range shifts driven by climatic changes are incorporated.

To improve spatial conservation planning one can map and quantify species' range shifts driven by climate change, measuring how much (and in which direction) a species is expected to move, and include this specific response in priority-setting analyses. For example, a species that is highly sensitive to changes in climate would either need (1) a larger conservation area compared to a less sensitive species or (2) the conservation of an area that is currently out of its geographic distribution. Species climate-forced dispersal takes place when some species need to disperse to sites that will become climatically suitable in the future because those in which they currently occur are becoming unsuitable.

Here we used consensual projections of ENMs to generate a nested ranking of priority sites for species conservation that considers species climate-forced dispersal by minimizing the distance a species would need to move to find a climatically suitable site. We measured uncertainty associated to ENMs and used it to minimize model uncertainties, favoring the inclusion of low-uncertainty sites in conservation plans. We also considered the current network of protected areas established in the region in our analyses. Hence our plans complement the level of protection already achieved in the region.

## Methods

### Geographic extent of the study

We focused our study in the Atlantic Forest. This natural domain is a Biodiversity Hotspot given its high level of plant endemism and a massive loss of it natural vegetation cover [17]. Originally extending over 1.5 million km^2^ along eastern Brazilian coast, now only *ca.*11% of its natural cover remains [18], and only 7.2% of its remaining habitats are strictly protected in Brazil (I–IV IUCN protected areas categories; [19]). Here we used the historical Atlantic Forest domain extension [20] to acquire information on species original climatic conditions.

### Ecological niche models

We gathered information on geographic distribution (extent of occurrence maps downloaded from iucnredlist.org/technical-documents/spatial-data) of 431 amphibian species inhabiting the Atlantic Forest. We used amphibians as our case study because they are the most threatened vertebrate group on Earth [21], being particularly sensitive to climate change [2]. They also need urgent conservation actions in the Neotropics [22]–[23].

**Figure 1:**
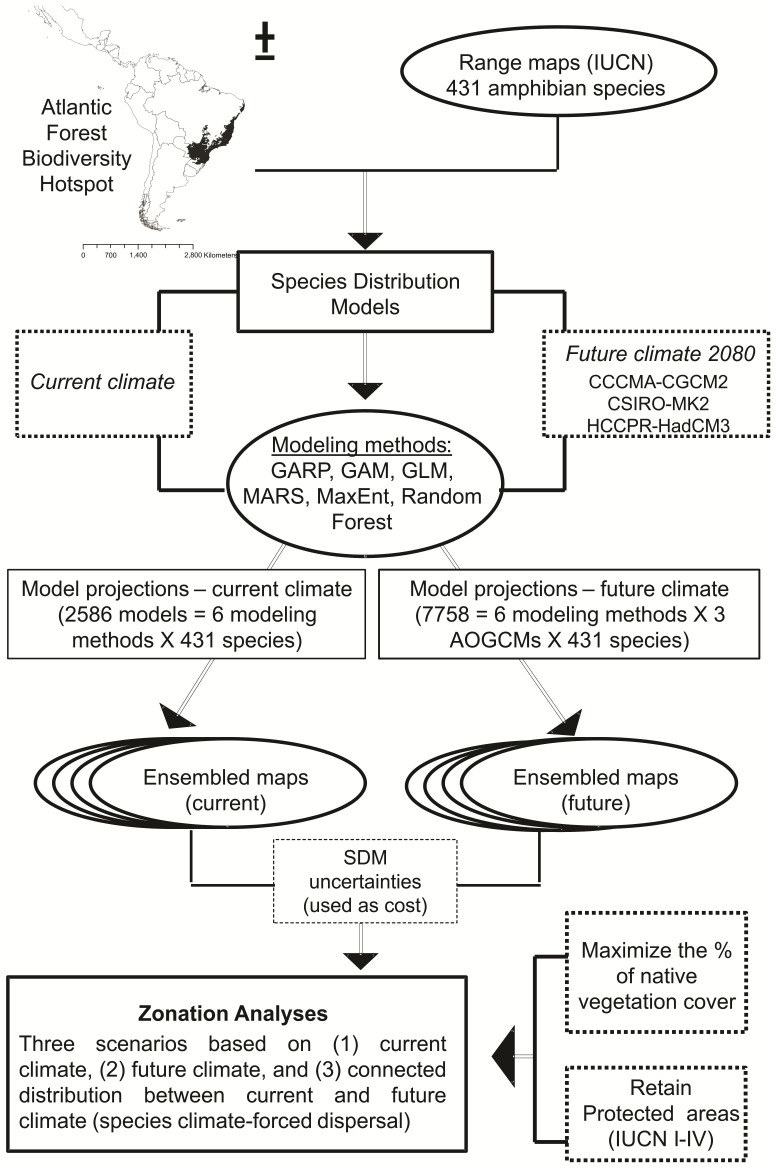
Flowchart of stages on analysis and data inputs used in this study. Flowchart of stages on analysis and data inputs used in spatial conservation planning approach designed to the Atlantic Forest Biodiversity Hotspot, Brazil, in the face of climate change.

Systematic conservation planning demands spatially extensive information on species distributions [24]. Although usually used, point location data are sparse and often biased in their sampling toward areas that are easily accessible, thus increasing omission errors. For these reasons, here we used digital range maps to generate a presence-absence matrix of amphibian occurrence in the Atlantic Forest. This matrix, along with climate variables (see bellow), was then used as our input data for building species' ecological niche models (Fig. 1). The use of range maps as input data to model species ecological niche is still incipient in the ENMs literature (but see Diniz-Filho et al. [13], Lawler et al. [25], for good examples). However, in regions of poor knowledge on species distribution, and under high threat to biodiversity, such approach may provide an initial identification of general priorities, which can be revised after data improvement [26]–[28].

We compiled current climatic data from the WorldClim database (worldclim.org/current), and future climatic scenarios from the International Center for Tropical Agriculture (CIAT, http://ccafs-climate.org), developed by IPCC's Fourth Assessment Report (AR4). We modeled species' ecological niche as a function of four climatic variables: annual mean temperature, temperature seasonality (standard deviation *100), annual precipitation, and precipitation seasonality (coefficient of variation). These variables are often used to explain patterns of amphibian species richness and distribution [29]. We used the following Atmosphere-Ocean Global Circulation Models (hereafter AOGCMs) projected to the mid-point of a 30-year period spanning 2071–2099 (i.e. 2080): CCCMA-CGCM2 – developed by the Canadian Centre for Climate Modeling Analysis, CSIRO-MK2 – developed by the Australia's Commonwealth Scientific and Industrial Research Organization, and HCCPR-HadCM3 – built by the Hadley Centre for Climate Predictions and Research's General Circulation Model. We choose these AOGCMs because they are widely used in the literature, having also different equilibrium climate sensitivity values ranging from 3.1°C to 4.4°C (see also Diniz-Filho et al. [13], Nori et al. [30]). Equilibrium climate sensibility is the annual mean surface air temperature change experienced by climate system after it has attained a new equilibrium in response to a doubling of CO_2_ concentration and are within the range of all AOGCMs available from International Panel on Climate Change (IPCC) [31].

We projected species' distribution in a 0.1×0.1 latitude-longitude grid (*ca.* 11 km size in the equator, totaling 11,461 equal-area grid cells). We used six modeling methods to built ENMs: Generalized Linear Models (GLMs [32]), Generalized Additive Models (GAMs [33]), Multivariate Adaptive Regression Splines (MARS [34]), Genetic Algorithm for Rule Set Production (GARP [35]), Random Forest (RF [36]), and Maximum Entropy (MaxEnt [37]). These methods are commonly used to generate ENMs, and details on each one of them can be found in Franklin [7]. For each species, data were randomly divided into calibration and validation sets, comprising 75 and 25% of the species' range, respectively. This procedure was repeated 50 times, maintaining the observed prevalence of species in each partition (i.e. for presence-only methods, 75% of the cells within the species' range, randomly defined; for presence-absence methods, we did the analyses using a random sample of 75% of cells both inside and outside species' range).

We established a threshold of pseudo-absences for each model to allow building the receiving operating curve (ROC) and transforming quantitative predictions of models into a binary vector of 0/1, indicating forecasted presences or absences in each grid cell [38]. We established the cut-off point by using the delimitation of bioclimatic envelope of 95%. We used True Skill Statistics (TSS) as our measure of model statistical fit. Sensitivity and specificity were calculated based on the probability threshold for which their sum is maximized, not being affected by prevalence. TSS values range from −1 to +1, where +1 indicates perfect fit, minimizing overprediction and omission error rates; values close to zero indicate performance worse than randomly expected [38]. We combined all model outputs generating ensemble-based frequencies of species distributions both for current and future climates. We considered species as occurring in a given cell if at least 50% of models predict its occurrence there (i.e. a majority consensus rule, see Diniz-Filho et al. [13]). Finally, we also calculated species turnover for each combination of modeling method, and AOGCM, which was based on the number of potential species gained (G) or lost (L) within each cell, and given by (G**+** L)/(S**+**G), where S is the species richness of the cell in the present [12],[39].

We averaged the projections of species distributions across each grid cell generating a species richness consensus map, as well as coefficients of variation that allow mapping where uncertainty in model projections is larger. To map uncertainties associated with SDMs, we did a two-way Analysis of Variance (ANOVA) without replication [40] to quantify variation associated to each source, using species richness as response variable and modeling methods and AOGCMs as factors [13]. We then obtained the sum of squares, which can be attributed to each of these sources. As we did the analyses for each grid cell covering the whole Atlantic Forest, it was possible to map each variance component and identify sites of low and high uncertainty [13]. We used the estimated proportion of the sum of squares attributable to the two sources in respect to total sum of squares (i.e. model uncertainty) as a constraint in spatial prioritization analyses (see below).

### Defining spatial conservation priorities for current time and for 2080

For solving the “utility maximization” problem we identified priority areas using the Zonation framework and software [41]–[42]. Zonation algorithm identifies sites primarily important for retaining high-quality and connected habitats for several features (e.g. species). It establishes a hierarchical ranking of conservation priorities for all cells throughout the geographic space, minimizing the loss of conservation value [41]. A cell is defined as “more important” when its relative contribution to total conservation value is the highest along the entire planning region. This level of importance is the conservation value of that cell.

Mathematically, the function for calculating the marginal loss (i.e. relative contribution of each cell to total conservation value) transforms the representation of feature *j* in site *i* into a general conservation value. There are different ways to calculate marginal loss of a given site *i* (δ*_i_*). The basic way is shown in equation 1:
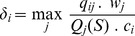
(1)where *q_ij_* is the representation level of feature *j* at site *i* (e.g. proportion of species' distribution in site *i*, in our case), *w_j_* is the weight (or priority) of feature *j*, and *c_i_* is the cost of site *i* (ENM uncertainty associated to site *i*, in our case). The term Q_j_(S) ∈ Σ_i_ Sq*_ij_*, is the proportion of original distribution of species *j* at the remaining set of sites S.

Here we used a variant of Zonation (the additive benefit function) that promotes representation of all species, favoring sites with high species richness while considering species' proportional distribution in a given cell [41]. Then, marginal loss of each site *i* was defined as a function of the sum for species-specific values that occur in the grid cell [i.e. summing the result of equation (1) for all species occurring at the cell, for all cells]. Here we calculate marginal loss using additive benefit function [43], as follows:
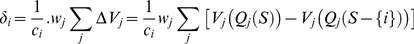
(2)In equation (2), marginal loss of cell *i* is simply the difference in conservation value of cell *i* found between the value (V*_j_*) in remaining set as a priority (S) and the value (V*_j_*) when the site *i* is removed from solution (S - {*i*}). Repeated iteration of equation (2) and removal of sites that generate the smallest loss of conservation value (i.e. smallest marginal loss) produce a rank based on complementarity, over the geographic space [42]. This rank is used to map priority sites for conservation. The last removed site is the one with the highest value of marginal loss, that is, the one that contributes the most to feature conservation (see Moilanen et al. [42], for more details). Note that this is a heuristic algorithm that does not necessarily achieve a solution that is optimal, but often near optimal. Yet, our problem is non-linear and very complex (with 431 species and >11,000 sites). In such cases, the degree of near-optimality associated to solutions is much less relevance given that the plan would not be implemented at once.

We established weights to species according to their conservation status defined by the IUCN Red List [37]: non-threatened species = 1, vulnerable and data deficient species = 2, endangered species = 3, critically endangered species = 4. All other species had weight = 1. Thus, weighting a critically endangered species as 4, means that maximizing representation of this species in priority sites is four times more important than doing so for a common species (because weights are multiplicative, see equation 2). The specific value of this weigh is arbitrary, although the transformation of IUCN Red List categories to an ordinal scale has been already used in conservation [45], as well as in spatial planning analyses (e.g. Gittleman [44], Loyola et al. [45]). Therefore, sensitivity analyses to test the importance of weighting would be required prior to utilization in a real-world conservation context.

Further, we included restricted protected areas (IUCN I-IV categories) already established in the Atlantic Forest (data from UNEP-WCMC [21]) in all spatial planning analyses (totaling 820 out of 11,461 grid cells). This means that our plans consider the current network of protected areas and indicate sites in which conservation investment should take place to complement the actual system.

In addition, we used information on the percentage of natural vegetation cover for each grid cell in all analyses as another attribute to be represented. We assigned weight five to this feature. We applied a weight for retaining natural vegetation higher than those for threatened species for two main reasons. First, given the lack of detailed information of habitat preferences for all species we studied, and the coarse spatial resolution of our vegetation layer (*ca.* 11 km), it was impossible to clip consensus species' distributions to natural vegetation remnants (which would diminish commission errors). Nevertheless, we controlled for model overprediction securing that only sites with currently available habitat to species survival and reproduction would be included in the spatial plan. Second, indicating that sites with high percentage of natural vegetation cover need to be retained means that every result indicates priority sites only in areas in which there are large remnants of natural vegetation to ensure real effectiveness of spatial plans, if they would be applied.

As explained above, we also used model uncertainties arising both from modeling methods and AOGCMs as a constraint in our analyses. This component is useful to assess the exclusion/inclusion effect of cell in priority site selection [46]. In our case, the estimated proportion of sum of squares attributable to the two sources in respect to total sum of squares, for each cell, was included as a constraint (or a cost, technically), c*_i_* (see equation 1 and 2). Including uncertainties in ENMs as a constraint means that sites for which there is low concordance among model projections produced by different modeling methods or climate models should not be prioritized because there is risk of misallocating scarce conservation resources in places where the certainty about the occurrence of species is low.

### Accommodating climate-forced dispersal in spatial conservation plans

Developing a dynamic spatial plan requires protecting important areas for conservation both in current and future climates. Here, assigned a high-conservation value only to sites that are suitable for each species both in current and future climates [41], because species distribution tends to be limited by contiguous suitable habitats [47]. For this, we obtained centroids of each species' distribution projected for present and future. Then, we assumed that Euclidean distance between current and future centroids of species' projected distribution could act as a measure of the dispersal ability of a species (in time).

In this case, the Euclidean distance between these centroids corresponds to a negative exponential function that describes species dispersal from present to future, forced by climate change [48], i.e. the distance that a species would need to move to find suitable climatic conditions in the future. We then used species-specific Euclidean distance between current and future distribution centroids to derive a bi-dimensional model estimated on the basis of the width of species-specific smoothing kernels (i.e. the distance a species would disperse under climatic changes, see Moilanen & Kujala [41]). Hence, the connectivity value among neighbor sites is directly proportional to occupancy level of species at the focal site. We used the distribution smoothing method available in Zonation to connect areas in agreement to surrounding area suitability. The method considers species-specific requirements in climate and dispersal capacity (based on smoothing kernels). The result is a set of priority sites that are more clumped in space [41]. There are few reasons for which aggregating sites at this spatial resolution would benefit species (especially considering extinction risk and metapopulation dynamics [41]), but fundamentally, this is the only way to include species climate-forced dispersal in analyses done here.

## Results

**Figure 2:**
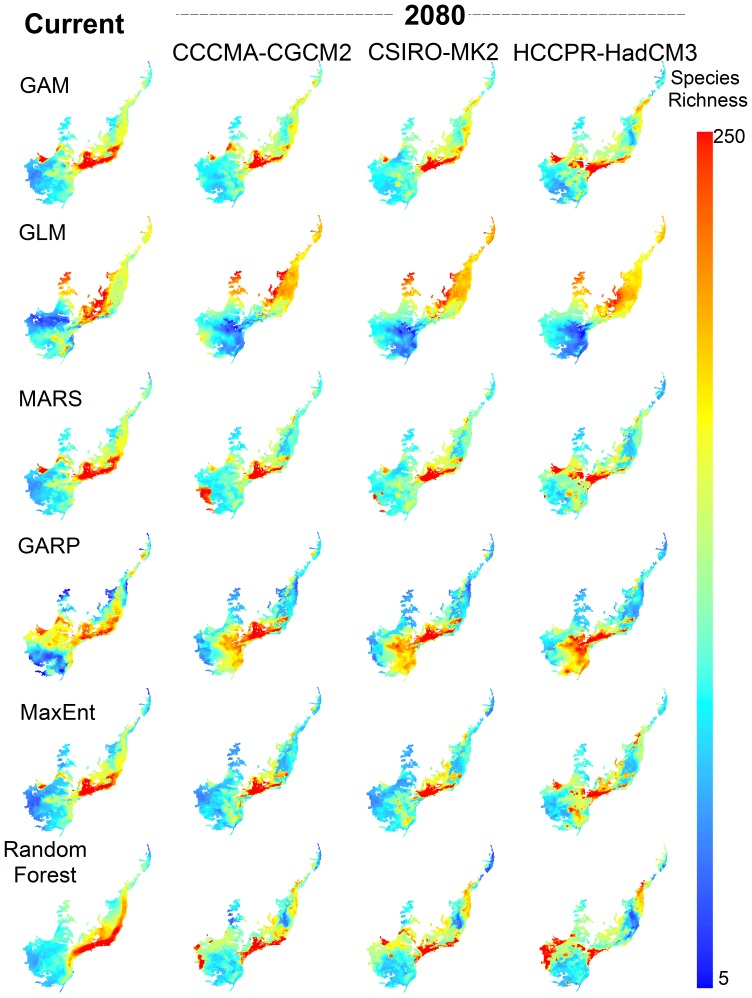
Different amphibian richness patterns for current and future climates in the Atlantic Forest, Brazil. Patterns of amphibian species richness projected by ecological niche models generated from different modeling methods (GLM, GAM, MARS, GARP, RF, and MaxEnt), and climate models (AOGCMs) both for current time and the year 2080 in the Atlantic Forest Biodiversity Hotspot, Brazil.

ENMs differed according to modeling methods and climate models used to project species' distributions (Fig. 2). Modeling methods were responsible for 72% of variation in projections, generating very distinct patterns of species richness. In general, all methods indicate high species richness in the eastern part of Atlantic Forest, with low species richness in southern and western portions of the biome. Projections of GLM provide a clear exception to this pattern, with richest areas concentrated in northeast, both for current time and for 2080 (Fig. 2). All models forecasted a general reduction in species' ranges, which leads to a decrease in the number of sites with high species richness. Variation among models projected into the future (i.e. within AOGCMs) was low, corresponding to only 0.5% of difference among maps (Fig. 2). For most species, TSS values were relatively high (TSS ± SD = 0.63±1.33) indicating good model fit.

**Figure 3:**
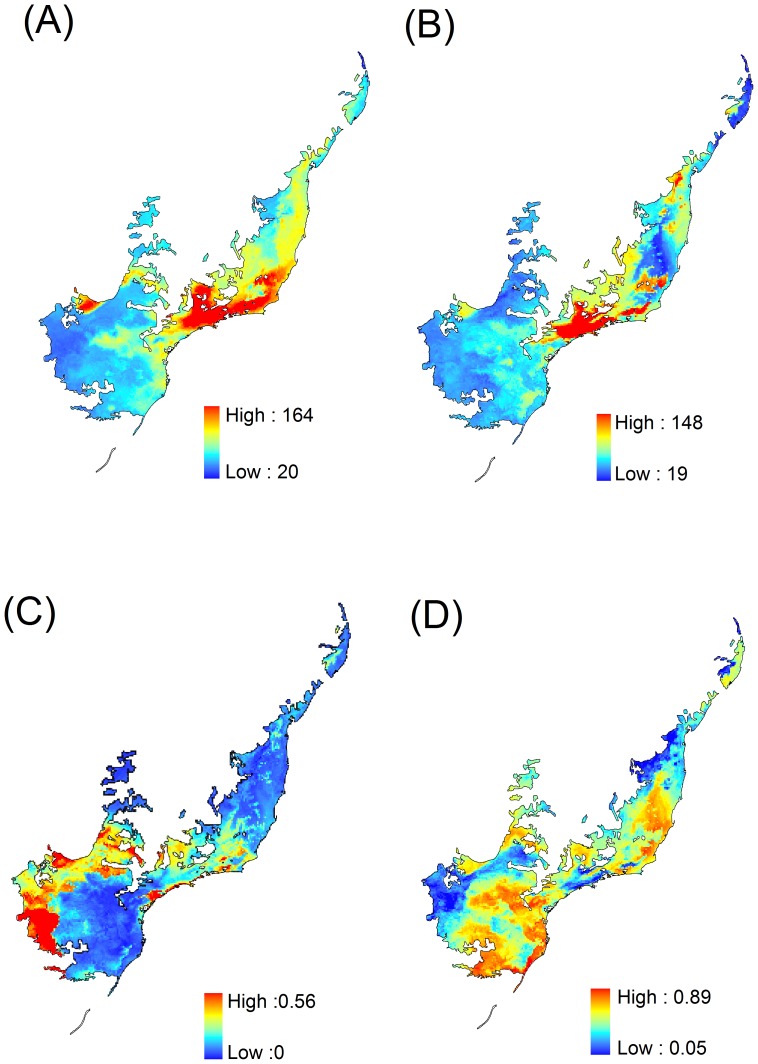
Consensus species richness patterns for current and future climates in the Atlantic Forest, Brazil. Consensus maps of amphibian species richness patterns for current time (A), and the year 2080 (B). Uncertainty level associated to ensemble of ecological niche models (C), and spatial patterns of species turnover (D) in the Atlantic Forest Biodiversity Hotspot, Brazil.

Hereafter, we will focus our attention in the consensus map, derived from the combination of all above-mentioned projections, weighted by their model fit (models with higher TSS value have more weight). Our consensus model forecasted range shifts that culminate with high species richness in central and eastern portion of the biome, both for current time and for 2080 (Fig. 3 A–B, respectively). Most species had a significant range contraction (up to 72%; mean ± SD = 38±2.38%) and 12% of species were projected to be regionally extinct. The western part of the biome is expected to have fewer species in the future.

The interaction between modeling methods and AOGCMs shows a different contribution to the geographically structured variation around consensus solution. ENM uncertainty is higher in southwest, but also along coastline (Fig. 3C). This means that, while there is no expected gain in species richness in the west, our models do not forecast the same effects of climate change in this particular region. Although future scenarios did not show dramatic changes in species richness, mean projected turnover was relatively high throughout the biome, ranging from 0.04 to 0.89. Southern and eastern Atlantic forest is expected to have high turnover rates (Fig. 3D).

For practical purposes, here we show the top 17% of cells that contribute the most to our conservation goal. The 17% of land area target was recently proposed for conservation of terrestrial ecosystems by the Convention on Biological Diversity [49]. It is a general target aimed at providing a fixed level of protection worldwide to be achieved by 2020. As it is a time-bound target recommended to encourage countries to increase their level of protected area coverage in the coming decade, coverage targets may be higher in the future. Hence, our proposed priority area network seeks only to meet the minimum target recommended for 2020 [49].

**Figure 4:**
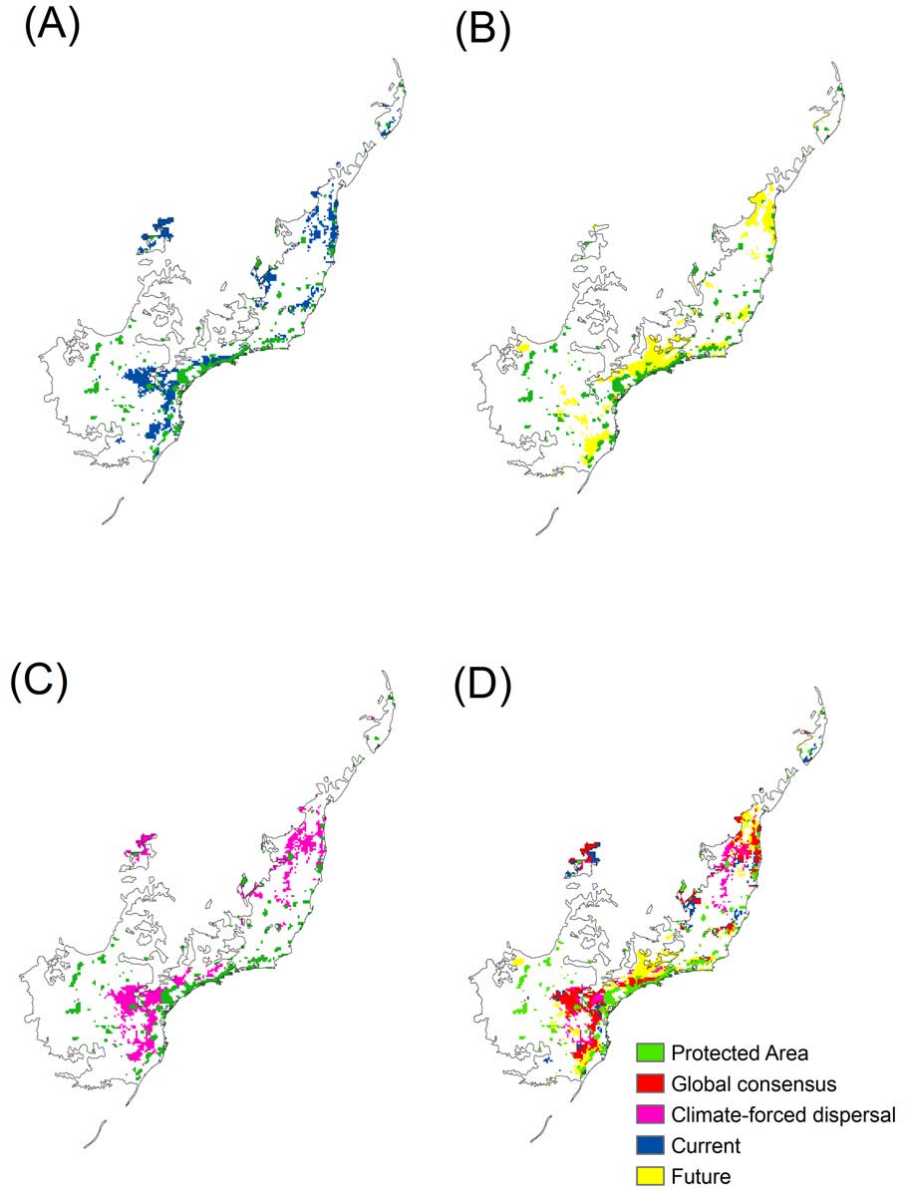
Priority sites for amphibian species conservation in the Atlantic Forest, Brazil. Top 17% of cell that should be prioritized if conservation of amphibian species inhabiting the Atlantic Forest Hotspot, Brazil, is planned for the present (A), future (B), and if we consider species climate-forced dispersal (from present to future, C). A full combination of these solutions is shown in Fig. 4D.

Priority sites for investment in amphibian conservation that complements current established network of protected areas differed when built upon present or future species distribution (Fig. 4A–B). We reached a compromised solution when we included species climate-forced dispersal in optimization procedure (Fig. 4C, compare similar areas in Fig. 4A and B). This later solution indicates a set of sites that are climatic suitable both in present and future, and that are connected by the likely dispersal distance species would be able to comprise during climatic changes. Hence, it already includes possible dispersal corridors linking current and future suitable climate in priority sites for amphibian conservation. Moreover, it also includes a minimization of errors associated with ENMs at remaining areas of native vegetation (Fig. 4C). A full combination of these solutions is shown in Fig. 4D.

Our results indicate that today we still need to protect at least an additional 9.8% of the biome to meet the 17% target (blue sites in Fig. 4A). If all priority sites proposed here only for the current time were to be converted in protected areas during the next 70 years we would reach such target. However, because of climate-driven species' range shifts, we would still need to protect an additional 1% of the Atlantic Forest to safeguard all amphibian species. For this reason, the solution presented in Fig. 4C is the best option among the ones we presented.

**Figure 5:**
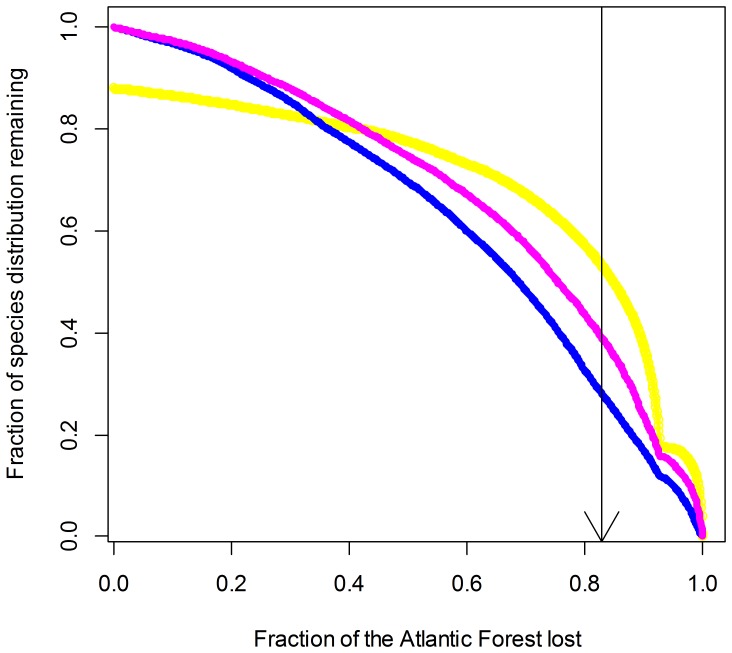
Performance curves of different spatial conservation solutions under climate change. Performance of conservation plans for amphibian species inhabiting the Atlantic Forest Hotspot, Brazil. Line colored in magenta represents the prioritization considering species climate-forced dispersal and model uncertainties. Blue and yellow lines stand, respectively, for the prioritizations based on current and future species distribution models. Colors as in Fig. 4.

In addition to priority maps, curves plotting the performance of solutions (Fig. 5) provide valuable insights on the relative protection attained under different climatic contexts. The figure shows the fraction of species distribution remaining against the fraction of remaining sites in the Atlantic forest, as the algorithm gradually eliminates cells with the smallest marginal loss. The arrow in Fig. 5 indicates the tipping point where 83% of the biome is lost (therefore retaining the best 17% of its land surface for protection).

## Discussion

Our analyses use a conservation biogeography approach [50] to evaluate changes in species' ranges as a function of climate change, and to optimize priorities sites for conservation under such threat. Instead of focusing on a particular species (e.g. an invasive species, Nori et al. [30]), we evaluated the efficiency of conservation plans as a function of environmental variables for a large number of species. We believe our results convey important recommendations for environmental management and policy. Although we used a top-ranked Biodiversity Hotspot and amphibians as a case study for illustrating our approach, we believe this could help developing effective conservation actions under a dynamic assessment (like those expected in face of climate change). Therefore, our approach may be applied to different spatial scales, geographic regions, and taxonomic groups.

Some authors showed greater robustness of consensual models when compared to a particular model [51]–[52]. The ensemble forecasting approach minimizes the difficulty of establishing the best criterion to evaluate performance of ecological niche models [10]. Moreover, the central tendency of selected forecasts has greater precision of species distribution since this consensus model covers a full range of uncertainties [51]. Notice that 72% of variation around of consensus model is due to different techniques used to model species' ecological niche and project their distribution. Moreover, it gives an objective measurement of uncertainty in the process, which can be mapped or, as done here, used to generate a weighting scheme in spatial conservation prioritization (see below).

Application of species' ENMs assumes that species exhibit an unlimited dispersal ability and absence of biological interactions [53]. Thus, our models result from the interaction between mechanisms operating at a broad spatial scale, given that species distributions are driven only by environmental or climatic conditions. Further, our modeling approach does not consider any changes in shape and size of the biome itself [54]. Likewise, we did not included neighboring species outside of biome that can immigrate to Atlantic Forest due to climate change. Finally, coarse scale data can over-exaggerate the impact of climate change on species distribution [55].

Our results show that most species will likely experience a significant range contraction in the future, and many others could face extinction. The contraction of a species' range is a reasonable concern since it increases the probability of species extinction [56]. The clearest impact should occur along the coastal line and the limits of the biome where we expect more changes in distribution. In fact, changes in species richness are not expected to be high in tropical regions given relatively short environmental gradients. However, given that species inhabiting the tropics may have narrower niches than their temperate counterparts, they are potentially susceptible to even small alterations in climatic conditions [57]–[58].

Although projections for the future cannot be truly validated given the dynamic processes of natural systems [6], ENMs still figure as the best strategy to obtain data that will be used into conservation planning [59]. Predicting the future is obviously not trivial, as it requires model extrapolation, so the effectiveness of conservation strategies will depend on suitable habitats for species both now and in the future [60]. Here, we addressed the problem of incorporating species range shifts (climate-driven dispersal) in spatial prioritization conservation. Our approach included the minimization of distance between centroid of the range maps in present and future. Spatial conservation plans will be obviously more effective if the effects of climatic changes can be anticipated [59]. Today, the number and location of networks of protected area are still mostly based on current species distributions [61]–[62], ignoring range shifts that should happen with climate changes.

Recently, some authors planned for species persistence over time, considering a dynamic environment and planning for reserve connectivity in fragmented landscape [4],[14]. Game et al. [63], for example, described strategies for climate change adaptation as part of national conservation assessment in Papua New Guinea. In particular they demonstrate that inclusion of climate refugia and cross-environment connectivity would make possible to reduce the amount of environmental change expected to take place inside protected areas [63]. We support their recommendations. For the Atlantic Forest, most remaining habitats figure as small and isolated forest remnants [17], which highlights the importance of connectivity between different habitats to accommodate species climate-forced dispersal. All this should be considered in future conservation assessments for the region.

The best 17% of the Atlantic Forest covers different proportions of species ranges when planning is made for different time periods, or to accommodate climate-forced dispersal. Within the best 17% of sites (see Fig. 5), the apparently inferior performance of the spatial solution based on species climate-forced dispersal (line colored in magenta) depends on the way marginal loss was calculated: it is constrained to select cells that may include combinations of site with both high and low conservation value, while trying to represent most of species distribution in conservation planning. Thus, analysis based only on future geographic distribution is apparently better in terms of species representation – because distributions are smaller. However, investing in conservation plans based only on future distribution models is problematic, since there is no guarantee that species will in fact shift their geographic range to the predicted location. Thus, we highlight the importance of the solution shown in Fig. 4C, which shows the climate-forced dispersal scenario. As expected, the performance of the climate-forced dispersal solution is intermediate between current and future solution, as it represents a compromise between these two.

Several sources of uncertainty arise in the process of conservation planning [64],[65]. Available species distribution data are incomplete or with high-commission errors (false presence) due to the interpolation of occurrence records [66]. Secondly, ecological niche modeling techniques introduce uncertainties, because model projections vary [13]. Of course, it is desirable to achieve a compromise between low ENM uncertainty and the conservation value of a given site [64]. In addition, other aspects of uncertainty can also be considered when proposing the establishment of new protected areas, such as extinction risk related to patch area [67]–[68] and availability of land for immediate acquisition [69]. Here we developed a more general conceptual model for establishing a dynamic spatial conservation prioritization analysis (see Fig. 1) that help planners to identify locations that are important both for the current time and for future scenarios of climate change. This is one of the top-priority questions in spatial conservation prioritization [70] and opens a strategy to the establishment of dynamic programs and conservation planning analyses that may help to better allocate scarce resources for biodiversity conservation [71]. We hope that our approach provides insights on the establishment of conservation priorities within sites of high biological importance in the face climate change.
